# Suppressive effects of the neutrophil elastase inhibitor sivelestat sodium hydrate on interleukin-1β production in lipopolysaccharide-stimulated porcine whole blood

**DOI:** 10.20407/fmj.2019-002

**Published:** 2019-11-02

**Authors:** Yasuyoshi Kurimoto, Yasuyo Shimomura, Kazuhiro Moriyama, Tomoyuki Nakamura, Naohide Kuriyama, Yoshitaka Hara, Hidefumi Komura, Daisuke Hasegawa, Takahiro Kawaji, Osamu Nishida

**Affiliations:** 1 Department of Anesthesiology and Critical Care Medicine, Fujita Health University, School of Medicine, Toyoake, Aichi, Japan; 2 Laboratory for Immune Response and Regulatory Medicine, Fujita Health University, School of Medicine, Toyoake, Aichi, Japan

**Keywords:** Sivelestat sodium hydrate, Acute respiratory distress syndrome, Myeloid cell, Cytokine

## Abstract

**Objective:**

Sivelestat sodium hydrate (Siv) is expected to be an effective therapy for acute respiratory distress syndrome, although its mechanism of action is not understood. In this study, we investigated which myeloid cells-derived cytokines were suppressed by Siv.

**Methods:**

Continuous hemofiltration was performed by circulating fresh porcine blood through a semi-closed circuit. To ensure that leukocytes survived for 360 min, 5% glucose, heparin, and air were continuously injected. The control group received continuous administration of lipopolysaccharide (LPS) only, whereas the Siv group received LPS and Siv. Complete blood count, levels of various cytokines, and other variables were compared between the groups.

**Results:**

Interleukin (IL)-1β level was significantly suppressed in the Siv group compared with that in the control group (p<0.05).

**Conclusions:**

The results suggested that Siv suppressed the production of IL-1β and possibly other cytokines by myeloid cells. Whether this suppression of cytokine production is caused directly by Siv or mediated via suppression of granulocyte elastase should be evaluated in the future.

## Introduction

Sivelestat sodium hydrate (Siv) is the first developed selective inhibitor of granulocyte elastase. Developed in Japan, it treats acute lung injury accompanied by systemic inflammatory response syndrome. In the field of intensive care, it is expected to be an effective therapy for acute respiratory distress syndrome (ARDS).^1,2^ Although Siv has recently been reported to suppress the production of cytokines and other mediators,^3,4^ its mechanism of action remains unclear. Cytokines are produced during inflammation for various reasons. Myeloid cells are known to be activated in ARDS.^5^ It is unknown whether the suppression of cytokine production by Siv occurs because of its action on myeloid cells. We hypothesized that Siv may suppress myeloid cell-derived cytokine production.

Previously, we constructed a system for maintaining the long-term viability of blood *ex vivo* by circulating fresh heparinized whole blood in a circuit containing a hemofilter to create an environment with fixed pH, electrolytes, temperature, oxygen partial pressure, carbon dioxide partial pressure, glucose, and other parameters inside the flask.^6^ Continuously injecting lipopolysaccharide (LPS) into this circuit and adding a column that selectively acts on activated myeloid cells (Adacolumn^®^; JIMRO Co., Gunma, Japan)^7^ promotes myeloid cell-derived cytokine production. The present study aimed to use a hemofiltration system that promotes myeloid cell-derived cytokine production to investigate whether Siv, a selective inhibitor of granulocyte elastase used to treat ARDS, suppresses the production of these cytokines.

## Methods

The primary objective of this study was to determine whether Siv administration suppresses myeloid cell-derived cytokine production.

### Collection and processing of fresh porcine blood

Fresh blood was collected from female pigs (2–5 years old, weighing 150–250 kg) (Meat Inspection Center, Toyota, Japan). Immediately after collection, 20,000 units/L heparin and 0.5 g/L glucose were added to the fresh porcine blood, which was then placed on ice.

### Apheresis circuit conditions

**Figure 1:**
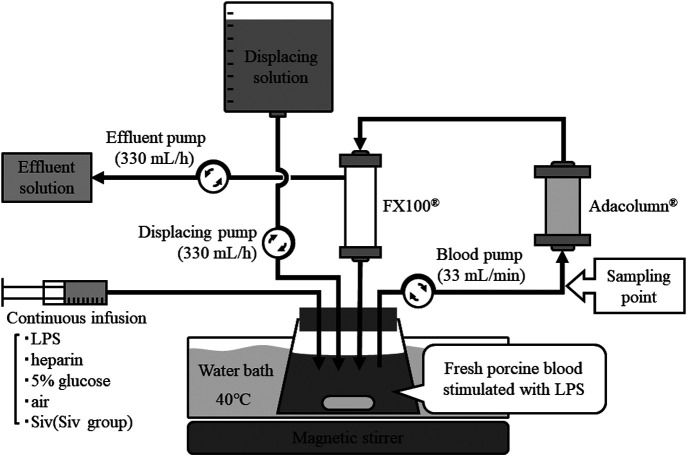
Schematic diagram of the blood preparation and treatment processes. Fresh porcine blood stimulated with LPS was maintained in a reservoir at 40°C. In addition to LPS, heparin, 5% glucose, and air were added to ensure that leukocytes survived for 24 h. The Siv treatment group received continuous administration of sivelestat sodium hydrate. The system was equipped with an Adacolumn^®^ (JIMRO Co. Ltd., Takasaki-shi, Gunma, Japan) and an FX100^®^ instrument (Fresenius Medical Care Japan, Tokyo, Japan). Continuous hemofiltration was performed to maintain electrolyte and pH levels. Blood was collected at the entrance of the primary column. CHF: continuous hemofiltration; LPS: lipopolysaccharide; Siv: sivelestat sodium hydrate.

To ensure that leukocytes survived for 24 h, 5% glucose at 1 mL/h, heparin at 2,000 units/h, and air at 0.5 L/min were continuously injected to the reservoir. To maintain constant electrolyte and pH levels, a semi-closed circuit was built to perform continuous hemofiltration (CHF). This semi-closed circuit was connected directly to an adsorptive myeloid cell apheresis column (Adacolumn^®^) (Figure 1). The FX100^®^ (Fresenius Medical Care Japan, Tokyo, Japan) and Adacolumn^®^ instruments were primed according to the instruction in their package inserts. The amount of blood circulating in the circuit was 1,300 mL. The CHF conditions were as follows: blood flow rate, 33 mL/min; filtration flow rate, 330 mL/h; and replacement flow rate, 330 mL/h.

### LPS injection

Three minutes after beginning circulation, an LPS bolus (*Escherichia coli* serotype 0111; Wako Pure Chemical Industries, Osaka, Japan) (5 mg/L) was injected to the circulation, followed by continuous LPS administration (0.22 mg/L/h). The amount of LPS was determined by referring to our previous research.^4^

### Group allotment and Siv administration

Some samples (the “Siv treatment” group) received a sivelestat (Elaspol^®^; Ono Pharmaceutical Co., Ltd., Osaka, Japan) bolus (154 mg/L) at 1 min before LPS administration, followed by continuous Siv administration (26 mg/L/h). The control group did not receive sivelestat bolus.

### Examinations

Blood samples for examination were collected at the entrance to the Adacolumn^®^ instrument before LPS injection and at 30, 60, 90, 120, 240, 300, and 360 min after LPS injection.

### Blood test

Complete blood count, biochemical tests (LSI Medience Corporation, Tokyo, Japan), and blood gas analysis were performed. Electrolyte, blood glucose, and lactic acid levels were measured.

### Cytokine measurement

A Bio-Plex multiplex system (Bio-Rad Laboratories, Tokyo, Japan) was used to measure IL-1β, IL-6, IL-8, TNF-α, IL-10, and IL-4 levels. An ELISA kit (Shino-Test Corporation, Tokyo, Japan) was used to measure the HMGB1 levels.

### Statistical analysis

Parametric data are expressed as mean ± standard deviation, whereas nonparametric data are expressed as median and interquartile range. Statistical analysis was performed by two-way analysis of variance (ANOVA). A probability level of <0.05 was used to indicate statistically significant differences. Stat Flex ver. 5 (Artech Corporation, Osaka, Japan) was used to conduct the statistical analyses.

### Ethics

The pigs were slaughtered in accordance with Act on Welfare and Management of Animals that was established by Ministry of the Environment. Our hospital’s ethical screening committee was consulted about performing the study using blood collected from pigs that were slaughtered for commercial purposes. The committee determined that there was no necessity for ethical screening.

## Results

The tests were conducted using five fresh porcine blood samples each for the Siv treatment and control groups (10 samples in total).

### Blood test results

No significant differences were observed between the Siv treatment and control groups in the biochemical and blood gas analysis results, nor in electrolyte, blood glucose, and lactic acid levels (data not shown).

### Blood count levels

**Figure 2:**
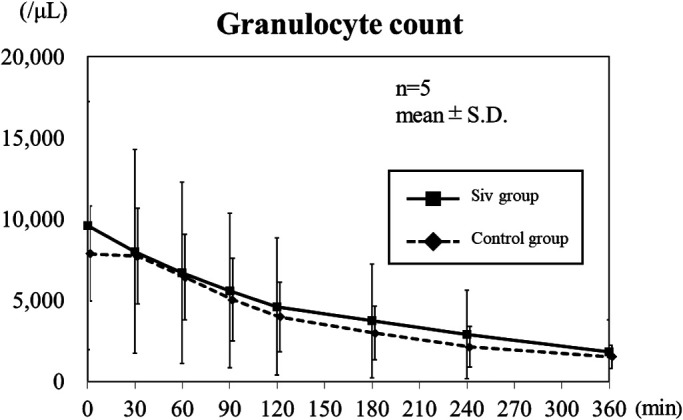
Granulocyte count in the Siv treatment and control groups. Granulocyte count decreased over time in both the Siv treatment and control groups. The difference in granulocyte count between the Siv treatment and control groups was not significant. S.D.: standard deviation; Siv: sivelestat sodium hydrate.

No significant differences were observed between the Siv treatment and control groups in erythrocyte, monocyte, and platelet counts (figures not shown). Regarding leukocytes, granulocyte count decreased over time in both the Siv treatment and control groups. However, the difference in granulocyte count between the Siv treatment and control groups was not significant (Figure 2).

### Cytokine levels

**Figure 3:**
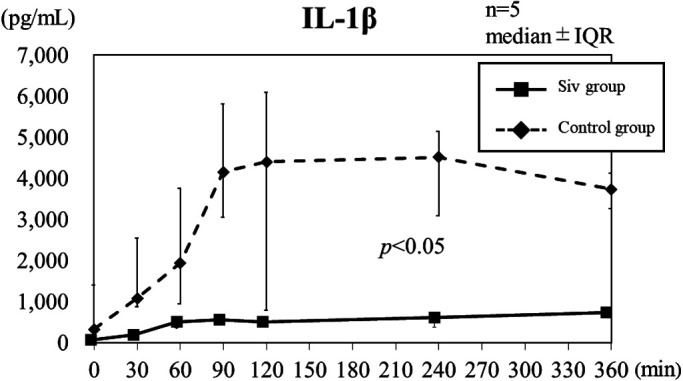
IL-1β level in the Siv treatment and control groups after LPS administration. In the control group, IL-1β level increased after LPS administration, and then remained high. In the Siv treatment group, IL-1β level remained low throughout. IQR: interquartile range; Siv: sivelestat sodium hydrate.

**Figure 4:**
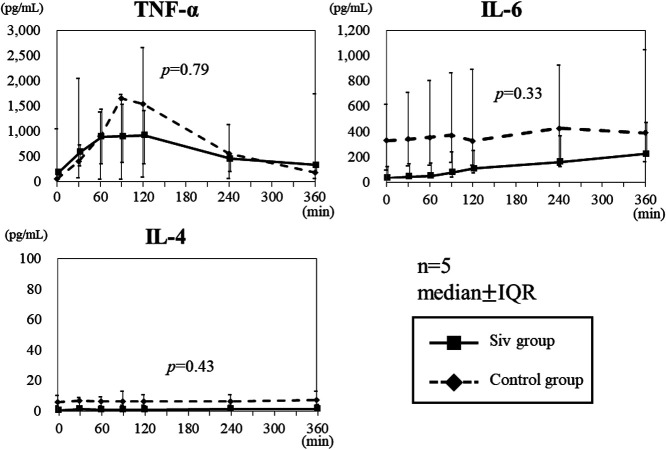
TNF-α, IL-4, and IL-6 levels in the Siv treatment and control groups. TNF-α peaked at a lower level in the Siv treatment group than that in the control group, although the difference was not significant. IL-6 level tended to be lower in the Siv group than in the control group, but the difference was not significant. IL-4 level was low in both the Siv and control groups, with no significant difference. There was a large dispersion in both groups. IQR: interquartile range; Siv: sivelestat sodium hydrate.

**Figure 5:**
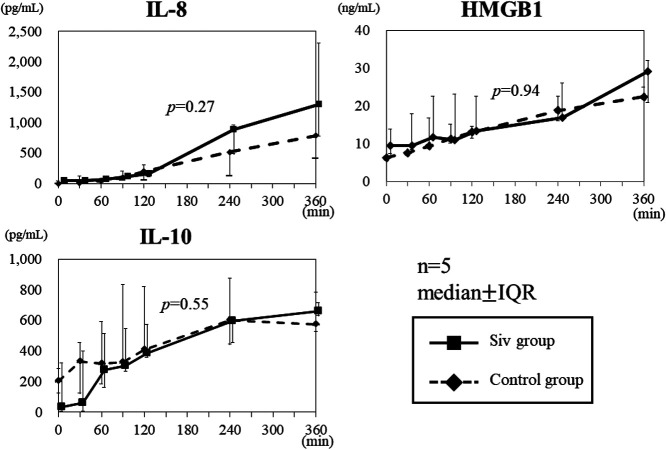
IL-8, HMGB1, and IL-10 levels in the Siv treatment and control groups. IL-8, HMGB1, and IL-10 levels tended to increase over time in both the Siv treatment and control groups. The difference in these levels between the Siv treatment and control groups was not significant. IQR: interquartile range; Siv: sivelestat sodium hydrate.

In the control group, IL-1β level peaked at 90–120 min after LPS administration, and then remained at a high level (Figure 3). In the Siv treatment group, IL-1β level increased over time, although all the values remained under 1,000 pg/mL. IL-1β level in the Siv treatment group was significantly suppressed, compared with that in the control group (p<0.05). The peak TNF-α level in the Siv treatment group was lower than that in the control group, although the difference was not significant (p=0.79; Figure 4). IL-4 and IL-6 levels in the Siv group were lower than those in the control group, but the differences were not significant (IL-4: p=0.33, IL-6: p=0.43; Figure 4). IL-8, IL-10, and HMBG1 levels increased with time in both the Siv treatment and control groups; however, these levels did not differ significantly between the Siv treatment and control groups (IL-8: p=0.27, IL-10: p=0.55, HMBG1: p=0.94; Figure 5).

## Discussion

Siv is a selective inhibitor of neutrophil elastase. However, its mechanism of action in ARDS is not understood. Previously, we constructed a continuous *ex-vivo* hemofiltration system that circulates fresh porcine blood in a hemofiltration circuit using the FX100 instrument, which maintained homeostasis of pH, electrolytes, and other parameters for 24 h.^6^

In the present study, we continuously injected LPS into this system and incorporated an Adacolumn^®^, which selectively adsorbs and eliminates myeloid cells (monocytes and granulocytes), to create an experimental system that promotes myeloid cell-derived cytokine production. When activated myeloid cells pass through the Adacolumn^®^, which is packed with cellulose acetate beads, they are adsorbed by the beads and eliminated.^7^ However, simultaneous to this adsorption, the activated myeloid cells also release cytokines.

This is an *ex-vivo* experimental system using blood that does not include the cytokines produced by organs in living organisms, which allowed us to focus only on hemocyte-derived cytokine production. Further, by using a column that selectively acts on myeloid cells, we believe the present study accurately reflected myeloid cell-derived cytokine production.

We compared the levels of seven types of cytokines, and the results showed that only IL-1β levels were significantly lower in the Siv group than in the control group. In addition, variation in TNFα levels was high in the control group, but low in the Siv group, thereby suggesting that increasing the sample size may reveal a significant suppressive effect. Moreover, IL-6 and IL-4 levels were low in the control group, which showed the difficulty in investigating the suppressive effects of Siv on cytokine production with this experimental system.

Although the suppressive effect of Siv on HMGB1 or IL-10 production was not observed, these cytokines are mediators secreted during the late inflammatory stage, which may be related to the results. One study examined the therapeutic effects of Siv in a rat sepsis model created by cercal ligation and puncture (CLP). The results showed that Siv administration improves survival rate and significantly reduces the serum levels of IL-1β, TNF-α, IL-6, and IL-10, although it did not reduce HMGB1 levels. However, the number of HMGB1-containing cells in lung tissues at 12 h after CLP was decreased by Siv administration.^8^

*In vivo* studies cannot determine whether cytokines with elevated levels are organ- or hemocyte-derived. The present study examined hemocyte components *ex vivo* to confirm that Siv suppressed myeloid cell-derived IL-1β production. However, measurements after 6 h are difficult owing to the mechanism by which Adacolumn^®^ column eliminates myeloid cells. Therefore, we could capture the changes in IL-1β level in the early inflammatory stage, but not in the cytokines with suppressed inflammation peaks after 6 h.

Another consideration regarding the suppressive effect of Siv on IL-1β is that unlike the secretion of the other six cytokines, IL-1β secretion accompanies the formation of inflammasomes. Inflammasomes, which are an important mechanism in the body’s inflammatory response to infection and other factors, secrete IL-1β and IL-18 via activated caspase-1.^9^ Elevation of the gene expression of IL-1β and IL-18 through the inflammasome/caspase-1 pathway has been reported in the peripheral blood of ARDS patients.^10^ Furthermore, in a study using vascular endothelial cells, IL-1β secretion was significantly reduced by inhibiting neutrophil elastase, not caspase-1.^11^ Taking the above findings into consideration, the results of the present study supported the hypothesis that the suppression of IL-1β production was part of the mechanism of the therapeutic effect of Siv on ARDS.

The cytokines IL-8, HMBG1, and IL-10 are produced during the late inflammatory stage. Therefore, the lack of significant differences in the results of our study may have occurred because the measurements were conducted only up to 360 min after LPS administration. Because the Adacolumn^®^ adsorbs and eliminates myeloid cells, they are almost completely eliminated after blood circulates in this circuit for 360 min, which is why we set 360 min as the measurement period in this study. It may be impossible to examine the effects of Siv on cytokine production after 360 min. In addition, heparin at 20,000 units/L was added to fresh porcine blood immediately after collection, which may have further affected the results. Moreover, in the present study, we used hemocytes and an *ex-vivo* experimental system with no organs; therefore, it is unclear whether the same results would be obtained *in vivo*.

In clinical practice, Siv is administered continuously at 0.2 mg/kg/h, which is equivalent to 2.6 mg/h of Siv per liter of circulating blood volume. The amount of blood circulating in our semi-closed circuit was 1,300 mL; thus, the continuously administered dose would be 3.38 mg/h. However, in the present study, Siv was injected at 154 mg/L and then administered continuously at 26 mg/h. Because Siv is likely to be metabolized by the liver, we adopted a higher dose than that used clinically. We plan to further study the dose-dependency of Siv’s effect.

A tendency toward suppressed TNF-α level was observed in the Siv treatment group, compared with that observed in the control group, but the difference was not significant. However, dispersion was large in the control group, suggesting that the lack of significant difference may have been due to the sample size. If the experiment had been performed with more than five samples in each group, we might have obtained significant differences. This was a limitation of the present study, and further studies with a larger sample size are needed to validate our results.

In conclusion, our findings suggested that Siv suppresses the production of IL-1β and possibly other cytokines by myeloid cells. Whether this suppression of cytokine production is caused directly by Siv or mediated through suppression of granulocyte elastase should be investigated in the future.
